# A Multicenter Retrospective Chart Review Study Estimating the Prevalence of Iron Deficiency Anemia Among Infants, Toddlers, and Children in Riyadh, Saudi Arabia

**DOI:** 10.7759/cureus.70031

**Published:** 2024-09-23

**Authors:** Ahmed M Almutairi, Sultan A Alwehaibi, Abdulaziz Y Almousa, Salma Alanazi, Rewaida R Bin Muaibed, Renad Althobaiti, Abdulaziz M Alkheraiji, Haya A Alamrani

**Affiliations:** 1 Pediatrics, College of Medicine, Imam Mohammad Ibn Saud Islamic University (IMSIU), Riyadh, SAU; 2 Medicine, College of Medicine, Imam Mohammad Ibn Saud Islamic University (IMSIU), Riyadh, SAU

**Keywords:** anemia, children, infants, iron deficiency, iron deficiency anemia, pediatric, prevalence, riyadh, saudi arabia, toddlers

## Abstract

Background

Iron deficiency anemia (IDA) is a prevalent nutritional disorder affecting children worldwide, leading to potential long-term cognitive and developmental deficits. This study aimed to determine the prevalence of IDA among healthy children attending Dr. Sulaiman Al-Habib Hospital Group in Riyadh, SAU, and explore the relationship between IDA and various demographic factors, including age, gender, and nationality.

Methodology

A retrospective chart review was conducted from July 2023 to August 2023 at Dr. Sulaiman Al-Habib Hospital Group, in Riyadh, Saudi Arabia. The study included 498 children aged 0.58 to 17 years, selected through a cluster sampling technique. Data on demographic characteristics and laboratory results, including hemoglobin (Hb) levels, mean corpuscular volume (MCV), mean corpuscular hemoglobin (MCH), mean corpuscular hemoglobin concentration (MCHC), red cell distribution width (RDW), ferritin, and RBC, were collected. Iron deficiency anemia was diagnosed based on Hb levels and other hematological indices. Statistical analysis was performed using SPSS Statistics version 23.0 (IBM Corp., Armonk, NY, USA).

Results

The prevalence of IDA among the study population was 9.2%, with 72.3% of the cases classified as mild and 27.7% as moderate. No cases of severe anemia were found. Infants had the highest prevalence of IDA (16.9%), followed by toddlers (12.0%), preschool children (7.2%), and school-age children (0.8%). Gender analysis revealed a prevalence of 9.7% in males and 8.6% in females, with no significant difference between genders (p = 0.659). All non-Saudi participants were free of IDA, whereas 9.3% of Saudi participants had IDA, though this difference was not statistically significant (p = 0.761).

Conclusion

The study found a relatively low prevalence of IDA among healthy children attending Dr. Sulaiman Al-Habib Hospital Group in Riyadh, SAU, with the highest prevalence observed among infants. These findings highlight the need for targeted interventions to prevent IDA, particularly in younger children. Routine screening and early treatment are crucial to mitigate the potential long-term effects of IDA on child development. Further research with larger and more diverse samples is recommended to validate these findings and explore additional factors influencing IDA prevalence.

## Introduction

Iron deficiency anemia (IDA) is one of the most common deficiencies in industrialized countries. At least 20% of the world’s population is deficient. Iron deficiency anemia (IDA) is currently estimated to affect more than 500 million people. The study by DeMaeyer and Adiels-Tegman showed that there was a striking relationship between anemia and both age and sex, with peak frequencies occurring in childhood and in pregnancy [1]. Iron is crucial for a baby's healthy development, particularly during the first two years of life when the central nervous system is developing [2]. The central nervous system is negatively affected by IDA. There are many liabilities associated with IDA in infants; the most important are impairments in psychomotor development and cognitive function [3]. Infants with severe IDA have shown lower cognitive test scores than infants with good iron status generally [1-4]. According to one study conducted in the Nablus Governorate, PAL, the prevalence of anemia among infants was approximately 34.6%, which was associated with multiple risk factors like maternal anemia during the third trimester, birth spacing less than three years, exclusive breastfeeding, and late introduction of complementary feeding [5]. The overall incidence of IDA in Saudi Arabia is not well established by epidemiological surveys; there are several reports from single institutions and for specific age or sex demographics, with reported prevalence ranging from 10% to 60% [6]. However, studies on the infant population are lacking. The objective of this study was to determine the current prevalence of IDA in Riyadh, SAU.

## Materials and methods

Setting

The study was conducted from July 2023 to August 2023 at Dr. Sulaiman Al-Habib Hospital Group in Riyadh, SAU. This retrospective chart review aimed to determine the prevalence of IDA among healthy children attending this hospital.

Selection of participants

Participants were selected using a cluster sampling technique from the Dr. Sulaiman Al-Habib Hospital Group. The study population included children presenting for outpatient visits from the beginning of 2020 until the end of 2023. A total of 1000 participants were listed, and a computer-based simple randomization was performed to select 500 eligible electronic medical charts. The final sample size was calculated to be 411 participants, considering an expected prevalence of 40%, a margin of error of ±5% with 95% confidence, and an anticipated 10% attrition rate.

Inclusion and exclusion criteria

The study included healthy children attending outpatient visits during the specified period. All infants (six months to one year), toddlers (one to three years), preschool children (four to six years), and school-age children (seven to 12 years) who underwent blood extraction at the Dr. Sulaiman Al-Habib Hospital Group were eligible for inclusion. Children were excluded if they had other causes of anemia or conditions contributing to low hemoglobin (Hb) concentration, such as chronic kidney disease, congenital heart disease, congenital anomalies, neurological diseases, endocrine abnormalities, liver diseases, and intestinal malabsorption. Additionally, children with a frequent or recent history of blood transfusion, acute infection, high C-reactive protein (CRP) levels (>5 mg/L), or high WBC counts were excluded. The WBC cutoffs were >17.5 x 10^9/L for infants and >13.5 x 10^9/L for preschool and school-age children.

Data collection

Data collection involved a retrospective chart review using a structured data collection sheet. The sheet consisted of two sections: the first section recorded demographic data (age, sex, nationality, and current weight), and the second section included biochemical profiles (Hb concentration, mean corpuscular volume (MCV), mean corpuscular hemoglobin (MCH), mean corpuscular hemoglobin concentration (MCHC), red cell distribution width (RDW), ferritin, and RBC count). Anemia was categorized by severity according to the WHO criteria: mild anemia (Hb 10-10.9 g/dL), moderate anemia (Hb 7-9.9 g/dL), and severe anemia (Hb <7 g/dL). Iron deficiency anemia was defined as an Hb level less than 11 g/dL with serum ferritin less than 10 µg/L. If ferritin was not available, IDA was defined by a combination of criteria: (1) Hb levels <11 g/dL, (2) MCV <70 fL, (3) RBC <3.5 x 10^12/L, (4) RDW index >220 (RDW index = (MCV)/RBC × RDW), (5) Mentzer index >13 (Mentzer index = (MCV)/RBC); Mentzer index is a screening tool to assist in differentiating between IDA and beta thalassemia trait (BTT). Iron deficiency anemia was confirmed if criteria 1 and 2 were met, along with at least one of criteria 3, 4, or 5 [7].

Data analysis

Data were analyzed using Microsoft Excel (Microsoft Corp., Redmond, WA, USA) and SPSS Statistics version 23.0 (IBM Corp., Armonk, NY, USA). Descriptive statistics were presented as numbers and percentages. The prevalence of IDA among the included children was calculated. The relationship between IDA and participants' sociodemographic characteristics was assessed, with a p-value of less than 0.05 considered statistically significant. Participants' data were treated with full confidentiality, ensuring that any published results would not be identifiable.

## Results

The study included 498 participants with an average age of 4.38 years (SD = 3.3 years), ranging from 0.58 to 17.00 years. The participants were categorized into four age groups: infants (24.9%), toddlers (23.5%), preschool children (27.7%), and school-age children (23.9%). The gender distribution was 55.6% male and 44.4% female. The overwhelming majority of the participants were Saudi nationals (98.6%), with only 1.4% being non-Saudi (Table 1).

**Table 1:** Demographic factors of the participants

Characteristics		Count	Percentage
Age (years)	Mean (SD)	4.38 (3.3)	
	Min-Max	0.58-17.00	
	Infant	124	24.9%
	Toddler	117	23.5%
	Preschool children	138	27.7%
	School-age children	119	23.9%
Gender	Male	277	55.6%
	Female	221	44.4%
Nationality	Saudi	492	98.6%
	Non-Saudi	7	1.4%

The laboratory results of the participants showed the following mean values: current weight was 19.10 kg (SD = 12.18 kg) with a range from 1.00 kg to 93.00 kg; Hb was 12.55 g/dL (SD = 1.98 g/dL) with a range from 7.80 g/dL to 35.00 g/dL; MCV was 81.65 fL (SD = 9.15 fL) with a range from 12.90 fL to 117.10 fL; MCH was 26.74 pg (SD = 4.34 pg) with a range from 12.50 pg to 80.90 pg; MCHC was 32.45 g/dL (SD = 2.10 g/dL) with a range from 22.70 g/dL to 37.00 g/dL; RDW was 12.88% (SD = 1.93%) with a range from 10.10% to 31.80%; ferritin was 39.09 ng/mL (SD = 40.04 ng/mL) with a range from 2.20 ng/mL to 311.55 ng/mL; and RBC was 4.84 x 10^12/L (SD = 2.32 x 10^12/L) with a range from 2.71 x 10^12/L to 55.00 x 10^12/L (Table 2).

**Table 2:** Laboratory results of the included participants Hb: Hemoglobin, MCV: Mean corpuscular volume, MCH: Mean corpuscular hemoglobin, MCHC: Mean corpuscular hemoglobin concentration, RDW: Red blood cell distribution width

Parameters	Mean	Standard deviation	Minimum	Maximum
Current weight	19.10	12.18	1.00	93.00
Hb	12.55	1.98	7.80	35.00
MCV	81.65	9.15	12.90	117.10
MCH	26.74	4.34	12.50	80.90
MCHC	32.45	2.10	22.70	37.00
RDW	12.88	1.93	10.10	31.80
Ferritin	39.09	40.04	2.20	311.55
RBCs	4.84	2.32	2.71	55.00

The prevalence of IDA among the participants was found to be 9.2%, with 46 children diagnosed with IDA out of the total 498 participants. The severity of anemia among those diagnosed with IDA was categorized as mild in 72.3% of the cases, moderate in 27.7%, and none of the cases were classified as severe. The analysis of factors associated with IDA revealed significant variations across different age groups. Infants had the highest prevalence of IDA at 16.9%, serving as the reference group. Toddlers had a lower prevalence of 12.0%, with a risk ratio of 0.66 (95% CI: 0.32-1.38) and a p-value of 0.275, indicating no significant difference compared to infants. Preschool children had an even lower prevalence of 7.2%, with a risk ratio of 0.38 (95% CI: 0.17-0.85) and a p-value of 0.018, showing a significant decrease in risk compared to infants. School-age children had the lowest prevalence of IDA at 0.8%, with a risk ratio of 0.042 (95% CI: 0.01-0.31) and a p-value of 0.002, indicating a significantly reduced risk of IDA compared to infants. Gender analysis showed that males had a prevalence of 9.7% for IDA, while females had a slightly lower prevalence of 8.6%. The risk ratio for females compared to males was 0.87 (95% CI: 0.47-1.62) with a p-value of 0.659, indicating no significant difference in the prevalence of IDA between genders. Regarding nationality, all non-Saudi participants were free of IDA, whereas 9.3% of Saudi participants had IDA. However, the difference was not statistically significant, with a risk ratio of 0.640 (95% CI: 0.03-11.38) and a p-value of 0.761 (Table 3).

**Table 3:** Factors associated with prevalence of IDA IDA: Iron deficiency anemia *A p-value < 0.05 is considered statistically significant.

Parameters		IDA					
		No		Yes		Risk ratio (95 % Confidence interval)	p-value
		Count	Percentage	Count	Percentage		
Age group	Infant	103	83.1%	21	16.9%	Reference	
	Toddler	103	88.0%	14	12.0%	0.66 (0.32-1.38)	0.275
	Preschool children	128	92.8%	10	7.2%	0.38 (0.17-0.85)	0.018*
	School-age children	118	99.2%	1	0.8%	0.042 (0.01-0.31)	0.002*
Gender	Male	250	90.3%	27	9.7%	Reference	
	Female	202	91.4%	19	8.6%	0.87 (0.47-1.62)	0.659
Nationality	Saudi	446	90.7%	46	9.3%		
	Non-Saudi	7	100.0%	0	0.0%	0.640 (0.03-11.38)	0.761

## Discussion

Overview

This study aimed to determine the prevalence of IDA among healthy children attending the Dr. Sulaiman Al-Habib Hospital Group in Riyadh. Additionally, the study explored the relationship between IDA and various demographic factors, including age, gender, and nationality. The findings provide valuable insights into the prevalence and distribution of IDA in this specific population, contributing to the existing body of knowledge and offering a basis for future interventions and public health strategies.

Prevalence of IDA

The prevalence of IDA in the study population was found to be 9.2%, with the highest among infants at a prevalence of 16.9% and the lowest among school-age children (0.8%). This prevalence is relatively low compared to other studies conducted in different regions. In a previous study conducted by Salah et al. to assess the frequency of IDA among infants aged between nine and 12 months and its associated risk factors in rural areas of Nablus Governorate, PAL, the authors showed that the prevalence of IDA among infants aged nine months and 12 months was 34.6% and 32.6%, respectively [5]. In addition, Al Hawsawi et al. showed the prevalence of IDA among infants aged between six and 24 months was 49% [8]. In a retrospective study conducted in Dubai, UAE, the authors reported that the prevalence of IDA among 1595 preschool children aged between one and five years old was 71.2% [9]. Moreover, our results are lower than reported in Latin American countries, where the prevalence of IDA was 70% of infants between six and 12 months and 45% of those between 12 and 24 months [10]. In Brazil, the prevalence was between 50% and 83% [11,12] and in KSA, the prevalence of one study was 55% among schoolgirls aged from seven to 14 years [13]. The lower prevalence observed in our study could be attributed to the healthcare facilities available at Dr. Sulaiman Al-Habib Hospital Group, which may ensure better nutritional status and health care for children. Additionally, the study's focus on healthy children might have excluded those with underlying conditions contributing to higher IDA prevalence.

Age and IDA

Age was significantly associated with the prevalence of IDA. Infants exhibited the highest prevalence (16.9%), which is consistent with the literature indicating that infants are particularly vulnerable to IDA due to rapid growth and insufficient iron intake from diet alone [14,15]. The significantly lower prevalence of IDA among school-age children (0.8%) suggests that as children grow, their diet likely becomes more diverse and richer in iron, reducing the risk of anemia [16,17]. This finding aligns with a study by Zimmermann and Hurrell, which highlights the importance of dietary diversity in preventing IDA among older children [18].

Gender and IDA

The analysis revealed no significant difference in IDA prevalence between males (9.7%) and females (8.6%), with a risk ratio of 0.87 (p = 0.659). This finding is consistent with several other studies that found gender to be a non-significant factor in the prevalence of IDA among children [19,20]. However, some other studies showed a significant difference between genders in the prevalence of IDA, where some showed a higher prevalence in males [21,22] and others in females [9,23]. The slight variation observed could be due to random variations rather than a true gender disparity in IDA risk.

Nationality and IDA

Our study also examined the prevalence of IDA in relation to nationality. Interestingly, none of the non-Saudi participants had IDA, while 9.3% of Saudi participants did. Although this difference was not statistically significant, it could indicate potential socioeconomic or dietary differences between Saudi and non-Saudi families. Non-Saudi families may have different dietary habits or access to different types of food, which could influence iron intake and absorption. However, the small sample size of non-Saudi participants (n = 7) limits the strength of this conclusion, and further research with a larger non-Saudi sample and genetic predisposition factors is needed to validate these findings.

Laboratory findings

The laboratory results provided further insights into the nutritional and health status of the participants. The mean Hb) level was 12.55 g/dL, which is within the normal range for children [24]. The other hematological parameters, such as MCV, MCH, MCHC, and RDW, also fell within normal ranges, suggesting overall good health status among the majority of the participants. The mean ferritin level was 39.09 ng/mL, indicating that most children had adequate iron stores. However, the wide range of ferritin levels (2.20 to 311.55 ng/mL) suggests variability in iron stores, possibly reflecting differences in dietary iron intake and absorption. The criteria used to define IDA in this study, including Hb levels, MCV, RBC count, RDW index, and Mentzer index, provide a comprehensive assessment, ensuring that the diagnosis of IDA is accurate and reliable.

Comparison with other studies

The prevalence and demographic associations observed in this study align with several other studies conducted in different regions. For example, a study in India reported a similar age-related trend in IDA prevalence, with younger children being more affected than older ones [25]. Another study in the United States found no significant gender differences in IDA prevalence, supporting our findings [26].

Public health implications

The findings of this study have important public health implications. The relatively low prevalence of IDA among the study population suggests that current healthcare practices at Dr. Sulaiman Al-Habib Hospital Group are effective in preventing IDA among healthy children. However, the higher prevalence among infants indicates a need for targeted interventions in this age group. Strategies such as promoting breastfeeding, iron supplementation, and dietary diversification could be beneficial. Moreover, the study highlights the importance of routine screening for IDA in pediatric populations, especially among infants and younger children. Early detection and treatment of IDA are crucial to prevent long-term cognitive and developmental deficits.

Strengths and limitations

One of the strengths of this study is the use of a well-defined sampling method and comprehensive diagnostic criteria for IDA, ensuring accurate and reliable results. Additionally, the study's focus on a specific population attending a reputable hospital group adds to the generalizability of the findings within similar healthcare settings.

However, the study also has limitations. The retrospective design may introduce selection bias, as the study included only children attending outpatient visits, potentially excluding those with more severe health conditions. Additionally, the small sample size of non-Saudi participants limits the ability to draw definitive conclusions about the impact of nationality on IDA prevalence.

## Conclusions

This study provides valuable insights into the prevalence and demographic factors associated with IDA among healthy children attending the Dr. Sulaiman Al-Habib Hospital Group in Riyadh. It found a relatively low prevalence of IDA overall, with the highest prevalence observed among infants. The findings highlight the need for targeted interventions among infants and underscore the importance of routine screening and early treatment for IDA in pediatric populations. Further research with larger and more diverse samples is needed to validate these findings and explore additional factors influencing IDA prevalence, such as genetic predispositions and dietary interventions.
